# Effect of Hypoxia on the Pathogenesis of Acinetobacter baumannii and Pseudomonas aeruginosa In Vitro and in Murine Experimental Models of Infection

**DOI:** 10.1128/IAI.00543-18

**Published:** 2018-09-21

**Authors:** María Luisa Gil-Marqués, María Eugenía Pachón-Ibáñez, Jerónimo Pachón, Younes Smani

**Affiliations:** a Clinic Unit of Infectious Diseases, Microbiology and Preventive Medicine, Institute of Biomedicine of Seville (IBiS), University Hospital Virgen del Rocío/CSIC/University of Seville, Seville, Spain; Georgia Institute of Technology School of Biological Sciences

**Keywords:** Acinetobacter baumannii, Pseudomonas aeruginosa, hypoxia, murine experimental models of infection, pathogenesis

## Abstract

Hypoxia modulates bacterial virulence and the inflammation response through hypoxia-inducible factor 1α (HIF-1α). Here we study the influence of hypoxia on Acinetobacter baumannii and Pseudomonas aeruginosa infections. *In vitro*, hypoxia increases the bactericidal activities of epithelial cells against A. baumannii and P. aeruginosa, reducing extracellular bacterial concentrations to 50.5% ± 7.5% and 90.8% ± 13.9%, respectively, at 2 h postinfection. The same phenomenon occurs in macrophages (67.6% ± 18.2% for A. baumannii at 2 h and 50.3% ± 10.9% for P. aeruginosa at 24 h). Hypoxia decreases the adherence of A. baumannii to epithelial cells (42.87% ± 8.16% at 2 h) and macrophages (52.0% ± 18.7% at 24 h), as well as that of P. aeruginosa (24.9% ± 4.5% in epithelial cells and 65.7% ± 5.5% in macrophages at 2 h). Moreover, hypoxia decreases the invasion of epithelial cells (48.6% ± 3.8%) and macrophages (8.7% ± 6.9%) by A. baumannii at 24 h postinfection and by P. aeruginosa at 2 h postinfection (75.0% ± 16.3% and 63.4% ± 5.4%, respectively). *In vivo*, hypoxia diminishes bacterial loads in fluids and tissues in animal models of infection by both pathogens. In contrast, mouse survival time was shorter under hypoxia (23.92 versus 36.42 h) with A. baumannii infection. No differences in the production of cytokines or HIF-1α were found between hypoxia and normoxia *in vitro* or *in vivo*. We conclude that hypoxia increases the bactericidal activities of host cells against both pathogens and reduces the interaction of pathogens with host cells. Moreover, hypoxia accelerates the rate at which animals die despite the lower bacterial concentrations *in vivo*.

## Abstract (precis)

Hypoxia modulates bacterial virulence and the inflammation response through hypoxia-inducible factor 1α (HIF-1α). Here we study the influence of hypoxia on Acinetobacter baumannii and Pseudomonas aeruginosa infections.

## INTRODUCTION

Several pathogens, including Escherichia coli, Pseudomonas aeruginosa, Salmonella enterica serovar Typhimurium, group A and B streptococci, Staphylococcus aureus, and Chlamydia pneumoniae, have been shown to regulate hypoxia-inducible factor 1α (HIF-1α) (1–6). The bacterial lipopolysaccharide has been reported to activate HIF-1α through toll-like receptor 4 in macrophages and neutrophils under normoxia (2, 7–10).

Hypoxia seems to have a protective role against bacterial infections. Thus, HIF-1α-deficient macrophages and polymorphonuclear leukocytes (PMN) affect the intracellular killing of group B streptococci and P. aeruginosa, respectively, *in vitro* (1, 9). In mice, HIF-1α-knockout (KO) keratinocytes induced the development of larger necrotic lesions and decreased the animals' capacity to clear group A streptococci by reducing the recruitment of neutrophils to the site of infection (11, 12), and HIF-1α knockdown by small interfering RNA (siRNA) reduced the resistance of mice to P. aeruginosa keratitis (9). Likewise, the use of mimosine, a HIF-1α agonist, can boost the ability of phagocytes and whole blood to kill S. aureus and can reduce lesion size in a murine model of skin infection (13).

However, the influence of hypoxia on infections with Gram-negative bacteria remains to be understood. We know that hypoxia impairs the innate immune functions of airway epithelial cells during P. aeruginosa infection, and the reduction of HIF-1α expression by siRNA in bronchial epithelial cells enhances the immune response (14). More specifically, hypoxia reduced the production of interleukin 6 (IL-6) by keratinocytes from that under normoxia (11). Moreover, HIF-1α deletion, but not HIF-1α isoform I.1, in T lymphocytes abolished the antibacterial effect of these cells (15, 16).

During infection, bacteria must adapt to heterogeneous environments (17–19). Oxygen levels in the foci of infection (<1%) are much lower than those in healthy tissues (2.5 to 9%) (20) due to increased oxygen consumption by immune cells and pathogens, along with decreased perfusion due to vascular dysfunction (21–23). Therefore, the microenvironment at the area of infection plays a crucial role in determining the outcome of the infection. Hypoxia modifies not only the host cells but also bacterial metabolism and virulence (5). In P. aeruginosa and Mycobacterium tuberculosis, the expression of virulence factors, such as alkaline protease, siderophores, and exotoxin A, is reduced by hypoxia (24, 25). However, hypoxia can also increase the production of alginate and the expression of the PA-I lectin/adhesin by P. aeruginosa, causing a disruption in the intestinal barrier and allowing exotoxin A to cross the epithelium (26, 27). Exposure to hypoxia also induces antibiotic resistance in P. aeruginosa by an alteration of efflux pump expression (28). Together, these studies demonstrate the complexity of HIF-pathogen interactions.

The aim of this study was to evaluate the effects of hypoxia (i) on A. baumannii and P. aeruginosa pathogenesis *in vitro*, with regard to bactericidal activity and bacterial adherence/invasion, (ii) on A. baumannii and P. aeruginosa pathogenesis in murine models of infection, with regard to survival and bacterial loads, and (iii) on the innate immune response *in vitro* and *in vivo*.

## RESULTS

### Hypoxia increases HIF-1α levels in epithelial cells and macrophages.

HIF-1α levels in cell lines after 6 and 24 h under hypoxia (1% O_2_) and normoxia (21% O_2_) were measured. In epithelial cells, HIF-1α levels were 2.69 times higher after 6 h in hypoxia than in normoxia (2,296.98 ± 157.74 pg/ml versus 853.63 ± 95.47 pg/ml [*P* < 0.001]) and were higher after 6 h than after 24 h under hypoxia (1,107.70 ± 96.08 pg/ml versus 592.27 ± 48.86 pg/ml [*P* < 0.01]). In macrophages, HIF-1α levels were 1.50 times higher after 6 h in hypoxia than in normoxia (331.64 ± 52.93 pg/ml versus 220.67 ± 11.87 pg/ml) and were higher after 6 h than after 24 h under hypoxia (223.59 ± 7.05 pg/ml versus 235.27 ± 9.31 pg/ml). No significant differences in HIF-1α levels were observed in normoxia between the different times points analyzed.

The marked increase in HIF-1α levels after 6 h under hypoxia (1% O_2_) defined the duration of hypoxia prior to infection for the *in vitro* and *in vivo* experiments.

### Hypoxia increases the bactericidal activities of epithelial cells and macrophages against A. baumannii and P. aeruginosa.

**FIG 1:**
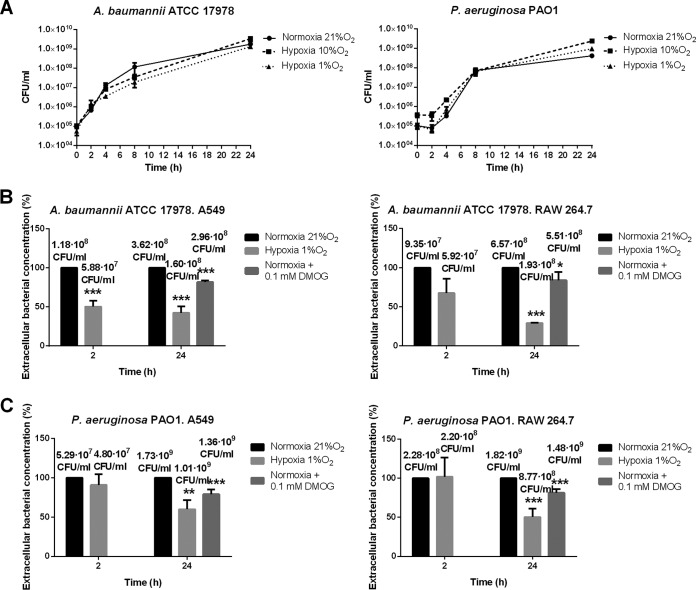
(A) Growth curves of A. baumannii ATCC 17978 and P. aeruginosa PAO1 under normoxia and hypoxia (10% and 1% O_2_). Three independent experiments were performed. (B and C) Measurement of bacterial concentrations (expressed as percentages) in the extracellular medium after 2- and 24-h infections of A549 and RAW 264.7 cells by A. baumannii strain ATCC 17978 (B) or P. aeruginosa strain PAO1 (C) under normoxia, hypoxia (1% O_2_), or normoxia plus treatment with 0.1 mM DMOG. Three independent experiments were performed. Asterisks indicate significant differences (***, *P* < 0.001; **, *P* < 0.01; *, *P* < 0.05) for hypoxia versus normoxia at 2 or 24 h and for normoxia plus DMOG versus normoxia with no treatment at 24 h.

First, we observed that the growth of strains ATCC 17978 and PAO1 for 2 and 24 h was indistinguishable between hypoxia (1% O_2_) and normoxia (Fig. 1A). Next, we asked if hypoxia affects the bactericidal activities of epithelial cells and macrophages. The counts of strains ATCC 17978 and PAO1 in the extracellular medium of both cell lines under hypoxia (1% O_2_) showed bacterial concentrations lower than those under normoxia after 2 and 24 h (Fig. 1B and C). These data support an increase in the bactericidal activities of these cell lines under hypoxia.

### HIF-1α overexpression increases the bactericidal activities of epithelial cells and macrophages against A. baumannii and P. aeruginosa.

Counts of strains ATCC 17978 and PAO1 in the extracellular medium of both epithelial cells and macrophages under treatment with 0.1 mM dimethyloxalylglycine (DMOG) showed bacterial concentrations lower than those under normoxia after 24 h (Fig. 1B and C). These data support an increase in the bactericidal activity of these cell lines when HIF-1α is overexpressed due to treatment with DMOG.

### Hypoxia decreases bacterial adherence to, and invasion of, epithelial cells and macrophages.

**FIG 2:**
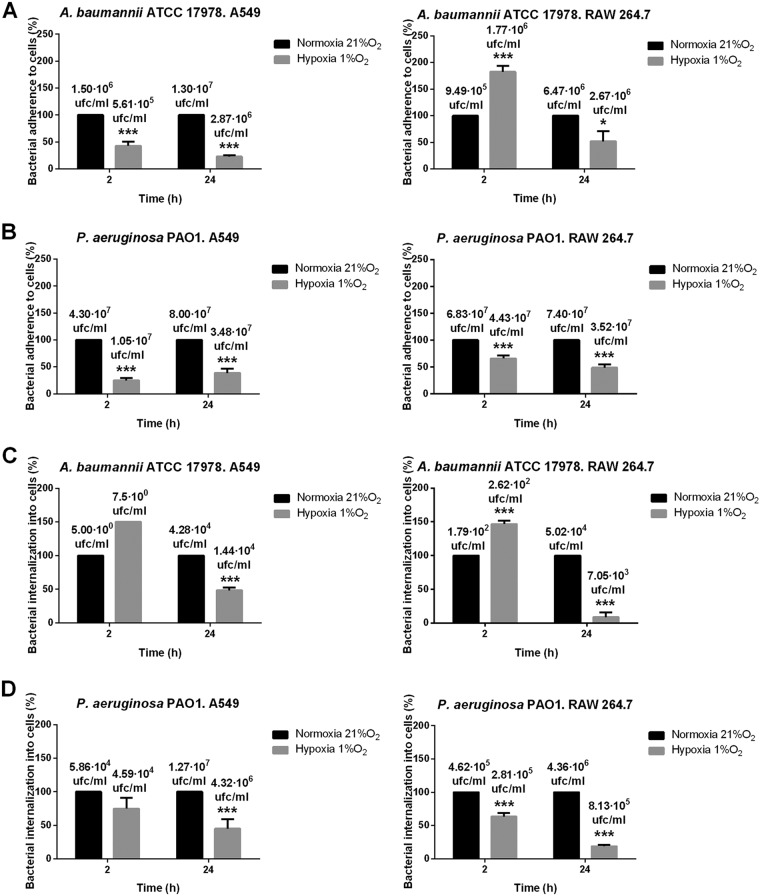
(A and B) Measurement of bacterial adherence (expressed as a percentage) after 2- and 24-h infections of A549 and RAW 264.7 cells by A. baumannii strain ATCC 17978 (A) or P. aeruginosa strain PAO1 (B) under normoxia or hypoxia (1% O_2_). Three independent experiments were performed. Asterisks indicate significant differences (*, *P* < 0.05; ***, *P* < 0.001) between hypoxia and normoxia at 2 or 24 h. (C and D) Measurement of bacterial internalization (expressed as a percentage) after 2- and 24-h infections of A549 and RAW 264.7 cells by A. baumannii strain ATCC 17978 (C) or P. aeruginosa strain PAO1 (D) under normoxia and hypoxia (1% O_2_). Four independent experiments were performed. Asterisks indicate significant differences (***, *P* < 0.001) between hypoxia and normoxia at 2 h or 24 h.

The adherence of strains ATCC 17978 and PAO1 to both epithelial cells and macrophages was significantly lower under hypoxia (1% O_2_), except for strain ATCC 17978 at 2 h postinfection in RAW 264.7 cells, which presented higher bacterial adherence (182.67% ± 11% versus 100% ± 0% [*P* < 0.001]) (Fig. 2A and B).

Counts of strain ATCC 17978 inside epithelial cells and macrophages under hypoxia (1% O_2_) showed increases in bacterial concentrations at 2 h postinfection (150% ± 0% for epithelial cells and 146.73% ± 5.01% for macrophages [*P* < 0.001]) and decreases at 24 h postinfection relative to concentrations under normoxia (48.55% ± 34.80% for epithelial cells [*P* < 0.001] and 8.6% ± 6.85% for macrophages [*P* < 0.001]) (Fig. 2C). On the other hand, counts of strain PAO1 inside both cell lines under hypoxia (1% O_2_) showed decreases in bacterial concentrations after 2 h (*P*, <0.001 for macrophages) and 24 h (*P* < 0.001) from concentrations under normoxia (Fig. 2D). These data indicated that hypoxia affects the adherence and invasion of A. baumannii and P. aeruginosa 24 h after bacterial infection.

### Hypoxia reduces the expression of proteins involved in cell adherence.

iTRAQ (isobaric tags for relative and absolute quantitation) results show that there are 51 downregulated proteins under hypoxia (fold change, <0.6) in the extracellular medium of A549 cells after a 2-h infection with A. baumannii strain ATCC 17978 (see Table S1 in the supplemental material). Forty-five percent are localized in the cytoplasm; 16% are secreted; 19% are in the inner membrane, 10% in the outer membrane, 6% in the periplasm, and 4% in the mitochondrion (see Fig. S1 in the supplemental material). The proteins localized in the outer membrane, which might be involved in cell adhesion, are OmpW, putative ferric siderophore receptor protein A1S_3339, putative ferric siderophore receptor protein A1S_0474, ferric enterobactin receptor A1S_0981, and ferrichrome-iron receptor A1S_1921. Moreover, the secreted uncharacterized protein A1S_3900, which presents SH3-like domains, might also be involved in cell adhesion.

### Hypoxia reduces bacterial loads in tissues and fluids in a peritoneal sepsis model with A. baumannii.

**FIG 3:**
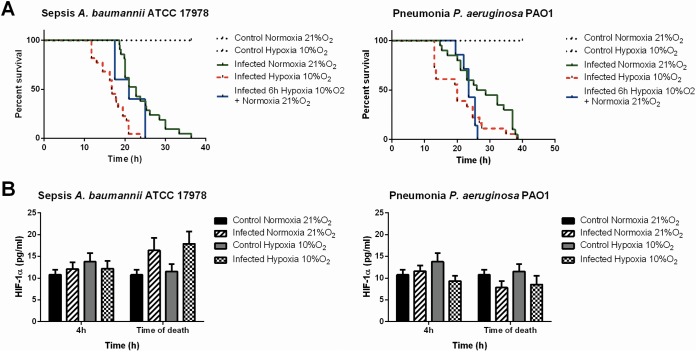
(A) Analysis of survival time under normoxia, hypoxia (10% O_2_), or 6 h of hypoxia (10% O_2_) followed by normoxia in the sepsis model with A. baumannii strain ATCC 17978 (*P*, <0.001 for hypoxia versus normoxia) and in the pneumonia model with P. aeruginosa strain PAO1 (*P*, <0.01 for hypoxia versus normoxia; *P*, <0.05 for 6 h of hypoxia followed by normoxia versus normoxia). (B) HIF-1α levels in mouse serum in the sepsis model with A. baumannii strain ATCC 17978 and in the pneumonia model with P. aeruginosa strain PAO1 at 4 h after infection and at the time of death under normoxia and hypoxia (10% O_2_).

The minimum lethal dose needed to achieve 100% mortality (MLD_100_) for strain ATCC 17978 was lower in hypoxia (10% O_2_) than in normoxia (2.08 log_10_ versus 3.20 log_10_ CFU/ml). For the rest of the experiments, we used the MLD calculated in normoxia. The survival time was higher for mice infected under normoxia than for those infected under hypoxia (10% O_2_) (36.42 versus 23.92 h [*P* < 0.001]) (Fig. 3A).

In the sepsis model with A. baumannii, regardless of the condition studied, all mice presented with bacteremia after 4 h of infection. No differences were found in the bacterial loads in tissues (spleen and lungs) and fluids (peritoneal fluid [PF] and blood) between hypoxia and normoxia after 4 of h infection (Table 1). However, at the time of death, significant differences between hypoxia and normoxia were found in the bacterial loads in lungs, PF, and blood (Table 1). Moreover, significant differences between animals under normoxia and those under hypoxia (6 h) followed by normoxia were found in the bacterial loads in the spleen, lungs, and PF at the time of death (Table 1). Bacterial loads in the spleen, lungs, PF, and blood were lower under hypoxia (either hypoxia for 6 h prior to infection or hypoxia during the whole experiment) than under normoxia.

**TABLE 1:** Bacterial loads in fluids and tissues in the sepsis model with A. baumannii strain ATCC 17978

Time and condition^*a*^	Bacterial load (log_10_ CFU per g of tissue or per ml of fluid) in:				Bacteremia (%)
	Spleen	Lungs	PF^*b*^	Blood	
4 h					
N	3.98 ± 0.30	4.07 ± 0.53	4.06 ± 1.29	3.19 ± 0.42	100
H	3.88 ± 0.23	4.07 ± 0.70	3.72 ± 1.15	3.18 ± 0.28	100
Time of death					
N	8.79 ± 0.56	9.36 ± 0.35	9.31 ± 0.33	8.40 ± 0.56	100
H	8.32 ± 0.71	8.25 ± 0.54^*e*^	8.88 ± 0.53^*c*^	7.73 ± 0.20^*d*^	100
H (6 h) + N	7.90 ± 0.30^*f*^	8.32 ± 0.46^*f*^	8.75 ± 0.33^*f*^	7.85 ± 0.32	100

a N, normoxia; H, hypoxia.

b PF, peritoneal fluid.

c *P* < 0.05 (H versus N at the time of death).

d *P* < 0.01 (H versus N at the time of death).

e *P* < 0.001 (H versus N at the time of death).

f *P* < 0.05 [H (6 h) + N versus N at the time of death].

HIF-1α levels showed no differences between hypoxia and normoxia in control animals (not infected). However, mice infected under the different conditions studied presented higher HIF-1α levels than control mice under normoxia at the time of death (Fig. 3B).

### Hypoxia reduces bacterial loads in tissues and blood in a P. aeruginosa pneumonia model.

The MLD calculated for strain PAO1 was the same for hypoxia and normoxia (8.54 log_10_ CFU/ml). Survival time was significantly higher under normoxia than under hypoxia (10% O_2_) (*P* < 0.01) or 6 h of hypoxia followed by normoxia (*P* < 0.05) (Fig. 3A). Pathological studies confirmed pneumonia 4 h after infection under all the conditions analyzed, but the symptoms were more severe under normoxia (data not shown).

After 4 h of infection, no significant differences in the bacterial loads in tissues and blood were found between the two conditions (Table 2). Nevertheless, at the time of the death of the mice, significant differences in the spleen, lungs, and blood were found between hypoxia and normoxia (Table 2). Similarly, significant differences were found in the bacterial loads in blood at the time of death between mice undergoing 6 h of hypoxia prior to infection followed by normoxia and mice infected in normoxia (Table 2). Bacterial loads in tissues and blood were lower under both hypoxemic conditions than under normoxia.

**TABLE 2:** Bacterial loads in fluids and tissues in the pneumonia model with P. aeruginosa PAO1

Time and condition^*a*^	Bacterial load (log_10_ CFU per g of tissue or per ml of fluid) in:			Bacteremia (%)
	Spleen	Lungs	Blood	
4 h				
N	2.64 ± 0.69	7.77 ± 0.61	0.26 ± 0.36	44.44
H	3.10 ± 0.80	7.79 ± 0.42	0.99 ± 0.84	61.11
Time of death				
N	6.96 ± 0.57	9.81 ± 0.45	7.90 ± 0.67	100
H	5.27 ± 0.60^*c*^	9.04 ± 0.58^*c*^	5.66 ± 0.78^*b*^	100
H (6 h) + N	6.60 ± 0.34	9.79 ± 0.27	6.30 ± 0.46^*d*^	100

a N, normoxia; H, hypoxia.

b *P* < 0.01 (H versus N at the time of death).

c *P* < 0.001 (H versus N at the time of death).

d *P* < 0.05 [H (6 h) + N versus N at the time of death].

HIF-1α levels did not differ significantly among the conditions studied. In contrast to the findings for the A. baumannii model of peritoneal sepsis, mice infected with P. aeruginosa under the different conditions studied presented lower HIF-1α levels than noninfected mice at the time of death (Fig. 3B).

### *In vitro* and *in vivo* cytokine production under hypoxia and normoxia.

**FIG 4:**
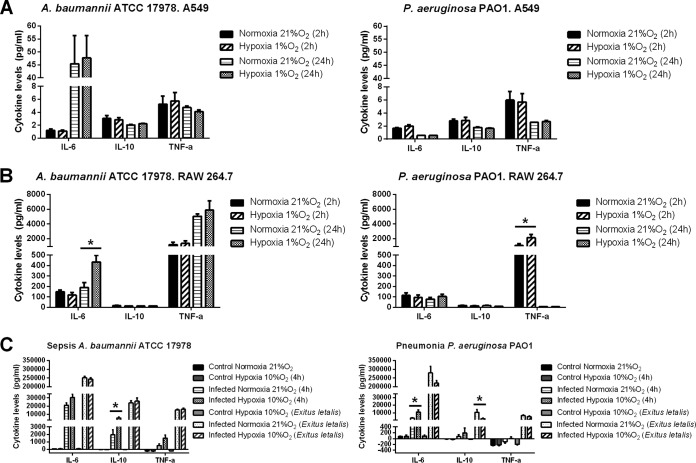
(A and B) Cytokine levels in the extracellular medium of A549 cells (A) or RAW 264.7 cells (B) infected with A. baumannii ATCC 17978 or P. aeruginosa PAO1 after 2 or 24 h under normoxia or hypoxia (1% O_2_). *, *P* < 0.05. (C) Cytokine levels in mouse serum in the sepsis model with A. baumannii strain ATCC 17978 and in the pneumonia model with P. aeruginosa strain PAO1.

Infection of epithelial cells by strains ATCC 17978 and PAO1 showed that IL-6, tumor necrosis factor alpha (TNF-α), and IL-10 levels were similar for hypoxia and normoxia at 2 and 24 h postinfection (Fig. 4A). When RAW 264.7 cells were infected with strains ATCC 17978 and PAO1, IL-6 levels at 24 h postinfection and TNF-α levels at 2 h postinfection were significantly higher in hypoxia (*P* < 0.05) (Fig. 4B).

In the sepsis model with strain ATCC 17978, only IL-10 levels were significantly higher after 4 h of infection under hypoxia (*P* < 0.05). No differences were found in IL-6 or TNF-α levels, although they were slightly higher under hypoxia. No differences in cytokine levels were found at the time of the animals' death (Fig. 4C). In the P. aeruginosa model of pneumonia with strain PAO1, IL-6 levels were significantly higher in hypoxia (*P* < 0.05) after 4 h of infection. Again, no differences were found for IL-10 and TNF-α levels, although they were rather higher under hypoxia (Fig. 4C). In contrast to what we observed in the sepsis model, at the time of the death of the mice, we observed lower IL-10 levels under hypoxia (*P* < 0.05). Again, no differences were found in IL-6 or TNF-α levels, although they were slightly lower under hypoxia (Fig. 4C).

## DISCUSSION

To our knowledge, this is the first study that analyzes the effect of hypoxia *in vitro* and *in vivo* during infection by A. baumannii and P. aeruginosa. We observed that hypoxia *in vitro* increases the bactericidal activities of host cells and reduces bacterial adherence and invasion. We also found that hypoxia *in vivo* diminishes bacterial loads in fluids and tissues but that mouse survival time is shorter under hypoxia.

We showed that hypoxia does not affect the growth of A. baumannii and P. aeruginosa *in vitro*. However, it increases the bactericidal activity in epithelial and macrophage cells. The study of Peyssonnaux et al. showed that hypoxia modifies gene regulation in host cells and increases levels of LL-37 cathelicidin, an antimicrobial peptide involved in the clearance of pathogens (12). Moreover, we see that hypoxia decreases bacterial adherence to host cells. This effect might be due to the modification of the cell or bacterial membrane under this condition. iTRAQ results confirmed that hypoxia downregulates 51 proteins in A. baumannii ATCC 17978, 5 of which are localized in the outer membrane and might be involved in cell adherence, in view of previous reports of their involvement in bacterial adherence (29–33).

Regarding bacterial invasion, we observed differences in behavior between A. baumannii and P. aeruginosa under hypoxia. Our data showed a reduction in P. aeruginosa internalization into epithelial cells and macrophages, confirming the results obtained in a previous study demonstrating that hypoxia decreases the internalization of P. aeruginosa into A549 cells (34). However, the levels of A. baumannii internalization in both types of host cells were higher after 2 h under hypoxia but were reduced after 24 h. Thus, hypoxia cannot stop A. baumannii invasion during the first few hours of infection, but invasion is finally hindered after 24 h. Therefore, we believe that hypoxia confers higher resistance against bacterial invasion on host cells in order to avoid intracellular replication and the evolution of infection.

In the *in vivo* experiments, we observe that a lower bacterial inoculum is needed to cause 100% mouse mortality under hypoxia in the peritoneal sepsis model with A. baumannii. All infected mice presented with bacteremia 4 h postinfection under all conditions studied. Moreover, we observe lower bacterial loads in the blood, PF, lungs, and spleen under hypoxia. We also show that maintaining animals under hypoxia for 6 h before the infection is enough to reduce bacterial loads at the time of death. These results are in accord with a previous study in which the use of the compound AKB-4924, which increases HIF-1α levels, reduced bacterial loads recovered in an S. aureus skin infection model (35). Moreover, these results are in accord with the data from *in vitro* adherence assays. The increase in the bactericidal activity of host cells under hypoxia, as well as the reduction in bacterial adherence, might allow the immune system to eliminate the infection better.

In the pneumonia model of infection by P. aeruginosa, we observed no differences in the inoculum needed to cause 100% mortality between the conditions studied. The levels of bacteremia observed in mice at 4 h postinfection were 44.44% and 61.11% for normoxia and hypoxia, respectively. The difference found in the levels of bacteremia between the two animal models is due to the severity of the sepsis model (36, 37). As in the A. baumannii sepsis model, we observed lower bacterial loads in fluids and tissues under hypoxia, and under hypoxia followed by normoxia, in agreement with the adherence and invasion results obtained *in vitro*. Again, as in the animal model of sepsis, the survival time was longer under normoxia.

In both animal models, HIF-1α levels were higher after 4 h under hypoxia, and the levels were similar at the time of animal death regardless of the conditions studied. These results are in agreement with those of the *in vitro* studies, in which HIF-1α levels increased over time under hypoxia and then decreased 24 h later. We observed that A. baumannii causes an increase in HIF-1α levels over those for the control, as reported in another study in which A. baumannii infection produced increases in HIF-1α levels (4). In contrast, P. aeruginosa reduces HIF-1α levels under hypoxia from those for the control. This result could be explained by 2-alkyl-4-quinolone and the Pseudomonas quinolone signal triggering HIF-1α degradation through the 26S proteasome proteolytic pathway, blocking the HIF-1α effect (38, 39).

As is well defined in the literature, hypoxia regulates the immune response (20). In the A. baumannii sepsis model, we observed high IL-10 levels under hypoxia after 4 h of infection. Meng et al. have indicated that HIF-1α is involved in IL-10 production by B cells (40), and IL-10 is an anti-inflammatory cytokine that suppresses macrophage and dendritic cell function (41). In the P. aeruginosa pneumonia model, we detected high IL-6 levels 4 h postinfection under hypoxia. It has been shown that HIF-1α increases TNF-α and IL-6 levels (20, 42). Moreover, IL-10 levels decreased under hypoxia in the P. aeruginosa pneumonia model at the time of death. However, we found no differences in cytokine levels between hypoxia and normoxia either *in vitro* or *in vivo*. Therefore, we found that hypoxia does not have a strong impact on cytokine production (20); its effects on the bactericidal activity of host cells and on the reduction of infection in animals are more important.

However, this study has some limitations. HIF-1α is a factor involved in multiple cellular pathways, and its expression is also regulated by different proteins. Therefore, finding a clear correlation between hypoxia, HIF-1α expression, inflammatory responses, and infection is complex, and multiple cellular processes have to be taken into consideration.

In conclusion, hypoxia increases the bactericidal activity of host cells. In contrast, mortality in animals under hypoxia is faster, even with lower bacterial loads in tissues and fluids, than under normoxia. Moreover, we find that hypoxia does not have a strong impact on cytokine production by either of the pathogens studied. Finally, although the two microorganisms studied are close phylogenetically, they present slightly different behaviors under hypoxia.

## MATERIALS AND METHODS

### Bacterial strains and growth conditions.

The wild-type strains A. baumannii ATCC 17978 and P. aeruginosa PAO1 were used. They were cultured at 37°C overnight (160 rpm) in Mueller-Hinton broth (MHB) (Sigma, Spain). Cultured strains were washed with phosphate-buffered saline (PBS) and suspended in Dulbecco's modified Eagle's medium (DMEM) before use in eukaryotic cell culture experiments (human lung epithelial cell line A549 and murine macrophage cell line RAW 264.7).

### Growth curve analysis.

The growth of A. baumannii ATCC 17978 and P. aeruginosa PAO1 under hypoxia (1% and 10% O_2_) and normoxia (21% O_2_) under static conditions was monitored for 24 h. Both strains were grown overnight in 20 ml of MHB, and a 1:10,000 dilution was performed to obtain approximately 10^5^ CFU/ml in a 40-ml culture of MHB (10% and 21% O_2_) or DMEM (1% and 21% O_2_). Three replicates were performed in different days.

### A549 and RAW 264.7 cell culture and infection.

The human lung epithelial cell line A549 and the murine macrophage cell line RAW 264.7 were grown in DMEM containing 10% fetal bovine serum (Gibco, Spain), 1 M 1% HEPES, vancomycin (50 mg/ml), gentamicin (20 mg/ml), and amphotericin B (0.25 mg/ml; Gibco), as described previously (43). In the case of hypoxia studies, cells were transferred to a hypoxia chamber (Coy Laboratories, USA) with a humidified atmosphere of 1% O_2_, 5% CO_2_, and the balance N_2_ at 37°C. Cells were seeded (10^5^/well in a 24-well plate) for 30 h in 24-well plates before infection with A. baumannii ATCC 17978 or P. aeruginosa PAO1 at a multiplicity of infection (MOI) of 500. To mimic a hypoxic condition, we treated the cells with 0.1 mM dimethyloxalylglycine (DMOG) (Sigma, Spain), an inhibitor of prolyl hydroxylases (44), 6 h prior to bacterial infection and during infection. Immediately before the infection, A549 cells were washed three times with PBS and were incubated in supplemented DMEM.

### HIF-1α measurement in cell cultures.

A549 and RAW 264.7 cells were seeded for 24 h in 6-well plates (10^6^ cells/well). After 6 and 24 h under hypoxia (1% O_2_) or normoxia, cells were washed three times with PBS, harvested using a cell scraper, homogenized in radioimmunoprecipitation assay (RIPA) buffer supplemented with 1 mM phenylmethylsulfonyl fluoride and a 10% cocktail of protease inhibitors (Sigma, Spain), and centrifuged at 13,000 × *g* and 4°C for 20 min. The supernatant was removed, and the amount of proteins was determined using the bicinchoninic acid (BCA) assay (Promega, Spain). The samples were stored at −80°C. Forty micrograms of proteins from each sample was used to measure HIF-1α levels with an enzyme-linked immunosorbent assay (ELISA) kit (Thermo Fisher Scientific, Spain).

### Bactericidal activity, bacterial adherence, and bacterial invasion in cell cultures.

After infections of A549 and RAW 264.7 cells with A. baumannii ATCC 17978 and P. aeruginosa PAO1 under hypoxic and normoxic conditions, the extracellular medium was removed and was serially diluted for the determination of bacterial concentrations as described previously (45).

Adherence and invasion assays were performed as described previously (45). To determine the number of adherent bacteria, cells were infected as mentioned above, and, after three washes with PBS, 200 μl of trypsin-EDTA (Gibco, Spain) was added for 5 min at 37°C. Then 200 μl of 0.5% Triton X-100 (Sigma, Spain) was added for 3 min. The invasion protocol included a treatment with gentamicin at 256 μg/ml (Gibco, Spain) before the addition of trypsin-EDTA. Diluted lysates were counted in order to determine the numbers of bacteria attached and internalized by A549 and RAW 264.7 cells.

Every assay was performed three times on different days. In the case of the invasion assay, four replicates were performed on different days.

### Cytokine assay.

The extracellular media of A549 and RAW 264.7 cells infected with A. baumannii ATCC 17978 or P. aeruginosa PAO1 under hypoxic or normoxic conditions were collected and centrifuged at 5,000 × *g* for 15 min at 4°C. The supernatant was stored at −80°C until analysis. TNF-α, IL-6, and IL-10 levels were measured using an ELISA kit (Affymetrix eBioscience, USA) in accordance with the manufacturer's instructions. Levels of pro- and anti-inflammatory cytokines (IL-6, IL-10, and TNF-α) in mouse serum were measured by ELISAs (Affymetrix eBioscience, USA).

### iTRAQ assay.

We analyzed the differential protein expression profiles under normoxic and hypoxic conditions in A549 cells infected with A. baumannii ATCC 17978. After 2 h of infection, we collected the cells in a lysis buffer composed of 1 M triethylammonium bicarbonate buffer (Sigma, Spain), 0.05% SDS, a 1:100 phosphatase inhibitor cocktail (PhosSTOP EASYpack; Roche, Spain), a 1:100 protease inhibitor cocktail (Complete Mini EDTA-free; Roche, Spain), and 0.002% Benzonase (Novagen, USA). The pellet was separated from the supernatant, and the protein concentration was quantified by fluorimetry (Qubit; Life Technologies, USA). Samples were treated with 50 mM Tris(2-carboxyethyl)phosphine hydrochloride (TCEP; AB Sciex, Spain) to reduce disulfide bonds and with 200 mM methyl methanethiosulfonate (MMTS; AB Sciex, Spain); then they were digested with trypsin (Promega, Spain) at a 10:1 substrate/enzyme ratio at 37°C overnight. We used iTRAQ 4-plex isobaric tags (with reporters at 114 to 117 Da; AB Sciex, Spain). Samples were analyzed by nano-liquid chromatography (nano-LC 100 system; Thermo Fisher Scientific, USA) and tandem mass spectrometry (MS-MS) (Q Exactive Plus Orbitrap system; Thermo Electron, USA). Proteins were identified using Proteome Discoverer, version 1.4 (Thermo Fisher Scientific, USA). MS-MS fragmentation patterns were mapped against the UniProt database. We considered quantifiable proteins to be those that were identified through >2 peptides with a confidence level of ≥95%, a *P* value of ≤0.05, and an error factor of <2 with every reference tag.

### Animals.

Immunocompetent C57BL/6 male mice, weighing approximately 20 g (Production and Experimentation Animal Center, University of Seville, Seville, Spain), were used; they had a sanitary status of murine pathogen free and were assessed for genetic authenticity. Mice were housed in an individually ventilated cage system under specific-pathogen-free conditions, and water and food were supplied *ad libitum*. This study was carried out according to the recommendations in the *Guide for the Care and Use of Laboratory Animals* (46) and in strict accordance with Directive 2010/63/EU on the protection of animals used for scientific purposes. Experiments were approved by the Committee on the Ethics of Animal Experiments of the University Hospital of Virgen del Rocío of Seville, Spain (20-05-14-84). All procedures were performed under sodium thiopental (B. Braun Medical S.A., Spain) anesthesia, and all efforts were made to minimize suffering.

### Experimental models.

Both models of infection were carried out under the following conditions: (i) hypoxia (10% O_2_), (ii) normoxia, and (iii) 6 h under hypoxia followed by normoxia. The MLDs were calculated for A. baumannii and P. aeruginosa under hypoxic and normoxic conditions. Briefly, groups of 6 mice were inoculated intraperitoneally (i.p.) for A. baumannii and intratracheally for P. aeruginosa with increasing concentrations of the pathogen until they reached 100% mortality, and the survival rate was monitored for 7 days. For the hypoxia studies, mice were maintained in a hypoxic chamber (Coy Laboratories, USA) with a humidified atmosphere of 10% O_2_ (standard hypoxic condition) 6 h prior to infection and until the death of the animal or the end of the experiment. In the experiments in which mice spent 6 h under hypoxia followed by normoxia, the animals were maintained in a hypoxic chamber during 6 h prior to infection and were placed outside under normoxia until the end of the experiment or the death of the animal. The same conditions were used for control mice (not infected).

To evaluate pneumonia, after 4 h of infection and at the time of death, lungs were aseptically extracted, fixed in 10% formalin, and embedded in paraffin wax. Serial sections (3 μm) were cut onto glass slides and were stained with hematoxylin and eosin.

Experiments with no more than 5 mice per condition were performed on different weeks to reproduce the experimental model results.

### (i) Experimental murine model of peritoneal sepsis.

A previously characterized murine model of A. baumannii peritoneal sepsis was used (36). Briefly, animals were inoculated i.p. with 0.5 ml of the MLD_100_, mixed 1:1 with a saline solution containing 10% (wt/vol) mucin from porcine stomach type II (Sigma, Spain).

After 4 h of infection, a group of 34 mice (17 under hypoxia and 17 under normoxia) were sacrificed by i.p. injection of sodium thiopental (200 μl; Braun Medical, USA) and were analyzed, and 48 mice (21 under normoxia, 22 under hypoxia, and 5 under 6 h of hypoxia followed by normoxia) were analyzed at the time of death. Survival rates were recorded under hypoxic and normoxic conditions. Bacteremia was evaluated, both qualitatively and quantitatively, after the animal's death. For qualitative analysis, the blood was inoculated into sterile tubes with 1 ml of MHB and was incubated for 24 h at 37°C, and then 10 μl was plated on sheep blood agar. To evaluate bacteremia quantitatively (as log_10_ CFU per milliliter), blood was serially diluted and plated on sheep blood agar. Finally, bacterial loads in the spleen and lungs were quantified. Briefly, organs were aseptically removed and homogenized (Stomacher 80; Tekmar Co.) in 2 ml of sterile 0.9% NaCl solution. Serial dilutions of the homogenized organs were plated on sheep blood agar for quantitative cultures (log_10_ CFU per gram). Finally, bacterial concentrations in peritoneal fluid were also determined by injecting 2 ml of sterile 0.9% NaCl solution i.p. and, after a brief massage on the abdomen, collecting peritoneal lavage specimens and plating them on sheep blood agar (log_10_ CFU per milliliter). HIF-1α levels in mouse serum were measured by ELISAs (MyBioSource, USA).

### (ii) Pneumonia model.

A previously characterized P. aeruginosa pneumonia model (47) was used as follows. Anesthetized mice (thiopental at 5% [wt/vol], i.p.) were infected by intratracheal instillation, using 50 μl of the MLD_100_ calculated earlier. Mice remained in a vertical position for 3 min and then were left resting in 30° positions until they awakened. After 4 h of infection, 36 mice (18 under normoxia and 18 under hypoxia) were sacrificed (with sodium thiopental; Braun Medical, USA) for analysis, and 46 mice (20 under normoxia, 18 under hypoxia, and 8 under 6 h of hypoxia followed by normoxia) were analyzed at the time of death. Survival rates were analyzed for the different conditions. Bacteremia levels and bacterial loads in blood and tissues (spleen and lungs) were determined as described above. HIF-1α levels in mouse serum were measured by ELISAs (MyBioSource, USA).

### Statistical analysis.

Statistical analyses were performed using the IBM SPSS Statistics 22 software program. Tests used included analysis of variance (ANOVA) (for bacterial counts in tissues and fluids and for time to mortality), the chi-square test (for bacteremia), and, when required, Dunnett's and Tukey's *post hoc* tests and Student's *t* test (for bacterial counts *in vitro*, cytokine levels, and HIF-1α levels). A *P* value of <0.05 was considered significant.

## Supplementary Material

Supplemental file 1
