# Supplementary material for: Effect of Hypoxia on the Pathogenesis of Acinetobacter baumannii and Pseudomonas aeruginosa In Vitro and in Murine Experimental Models of Infection
Source: Infect Immun. 2018 Sep 21;86(10):e00543-18. doi: 10.1128/IAI.00543-18 (PMC6204731; doi:10.1128/IAI.00543-18)
Supplement: Supplemental file 1 [file zii999092562s1.pdf]

SUPPLEMENTAL DATA

Table S1. Identification of the subexpressed proteins under hypoxia condition (1%O_2).

Accession	Description	Fold Change	Localization
A3M753	Putative outer membrane protein OmpW OS=Acinetobacter baumannii (strain ATCC 17978 / CIP 53.77 / LMG 1025 / NCDC KC755 / 5377) GN=A1S_2325 PE=4 SV=2 - [A3M753_ACIBT]	0,38	Outer membrane
A3M6R9	DNA-binding protein OS=Acinetobacter baumannii (strain ATCC 17978 / CIP 53.77 / LMG 1025 / NCDC KC755 / 5377) GN=A1S_2186 PE=4 SV=1 - [A3M6R9_ACIBT]	0,39	Cytoplasm
A3M6Q5	30S ribosomal protein S18 OS=Acinetobacter baumannii (strain ATCC 17978 / CIP 53.77 / LMG 1025 / NCDC KC755 / 5377) GN=rpsR PE=3 SV=1 - [RS18_ACIBT]	0,44	Cytoplasm
A3M178	Uncharacterized protein OS=Acinetobacter baumannii (strain ATCC 17978 / CIP 53.77 / LMG 1025 / NCDC KC755 / 5377) GN=A1S_0191 PE=4 SV=2 - [A3M178_ACIBT]	0,45	Secreted
A7FBY7	Uncharacterized protein OS=Acinetobacter baumannii (strain ATCC 17978 / CIP 53.77 / LMG 1025 / NCDC KC755 / 5377) GN=A1S_3900 PE=4 SV=2 - [A7FBY7_ACIBT]	0,45	Secreted
A3M3Z7	Benzoate transporter OS=Acinetobacter baumannii (strain ATCC 17978 / CIP 53.77 / LMG 1025 / NCDC KC755 / 5377) GN=A1S_1211 PE=4 SV=2 - [A3M3Z7_ACIBT]	0,45	Inner membrane
A3M4R4	Putative membrane protein OS=Acinetobacter baumannii (strain ATCC 17978 / CIP 53.77 / LMG 1025 / NCDC KC755 / 5377) GN=A1S_1480 PE=4 SV=2 - [A3M4R4_ACIBT]	0,46	Inner membrane
A3M9Y4	Putative ferric siderophore receptor protein OS=Acinetobacter baumannii (strain ATCC 17978 / CIP 53.77 / LMG 1025 / NCDC KC755 / 5377) GN=A1S_3339 PE=3 SV=2 - [A3M9Y4_ACIBT]	0,46	Outer membrane
A3M6W6	Uncharacterized protein OS=Acinetobacter baumannii (strain ATCC 17978 / CIP 53.77 / LMG 1025 / NCDC KC755 / 5377) GN=A1S_2233 PE=4 SV=1 - [A3M6W6_ACIBT]	0,46	Cytoplasm
A7FBL2	Uncharacterized protein OS=Acinetobacter baumannii (strain ATCC 17978 / CIP 53.77 / LMG 1025 / NCDC KC755 / 5377) GN=A1S_3775 PE=4 SV=2 - [A7FBL2_ACIBT]	0,47	Cytoplasm
A3M745	Putative lipoprotein (RlpA-like) OS=Acinetobacter baumannii (strain ATCC 17978 / CIP 53.77 / LMG 1025 / NCDC KC755 / 5377) GN=A1S_2317 PE=3 SV=2 - [A3M745_ACIBT]	0,47	Secreted
A7FBU1	Uncharacterized protein OS=Acinetobacter baumannii (strain ATCC 17978 / CIP 53.77 / LMG 1025 / NCDC KC755 / 5377) GN=A1S_3854 PE=4 SV=2 - [A7FBU1_ACIBT]	0,49	Inner membrane
A7FAY0	Uncharacterized protein OS=Acinetobacter baumannii (strain ATCC 17978 / CIP 53.77 / LMG 1025 / NCDC KC755 / 5377) GN=A1S_3543 PE=4 SV=2 - [A7FAY0_ACIBT]	0,49	Inner membrane
A3M9A7	Putative ATP-dependent RNA helicase OS=Acinetobacter baumannii (strain ATCC 17978 / CIP 53.77 / LMG 1025 / NCDC KC755 / 5377) GN=A1S_3104 PE=3 SV=1 -	0,49	Cytoplasm

	[A3M9A7_ACIBT]		
A3M4B3	FAD-dependent pyridine nucleotide-disulphide oxidoreductase OS=Acinetobacter baumannii (strain ATCC 17978 / CIP 53.77 / LMG 1025 / NCDC KC755 / 5377) GN=A1S_1329 PE=4 SV=1 - [A3M4B3_ACIBT]	0,49	Cytoplasm
A7FAV9	Uncharacterized protein OS=Acinetobacter baumannii (strain ATCC 17978 / CIP 53.77 / LMG 1025 / NCDC KC755 / 5377) GN=A1S_3522 PE=4 SV=2 - [A7FAV9_ACIBT]	0,49	Secreted
A3M2E7	Uncharacterized protein OS=Acinetobacter baumannii (strain ATCC 17978 / CIP 53.77 / LMG 1025 / NCDC KC755 / 5377) GN=A1S_0642 PE=4 SV=2 - [A3M2E7_ACIBT]	0,49	Cytoplasm
A3M9R3	Uncharacterized protein OS=Acinetobacter baumannii (strain ATCC 17978 / CIP 53.77 / LMG 1025 / NCDC KC755 / 5377) GN=A1S_3268 PE=4 SV=2 - [A3M9R3_ACIBT]	0,50	Secreted
A3M5K5	Putative MFS family drug transporter OS=Acinetobacter baumannii (strain ATCC 17978 / CIP 53.77 / LMG 1025 / NCDC KC755 / 5377) GN=A1S_1772 PE=4 SV=2 - [A3M5K5_ACIBT]	0,51	Inner membrane
A3M4T1	Putative acyltransferase OS=Acinetobacter baumannii (strain ATCC 17978 / CIP 53.77 / LMG 1025 / NCDC KC755 / 5377) GN=A1S_1497 PE=4 SV=2 - [A3M4T1_ACIBT]	0,51	Cytoplasm
A3M5H9	Putative transcriptional regulator OS=Acinetobacter baumannii (strain ATCC 17978 / CIP 53.77 / LMG 1025 / NCDC KC755 / 5377) GN=A1S_1746 PE=4 SV=2 - [A3M5H9_ACIBT]	0,51	Cytoplasm
A3M2X6	Putative signal peptide OS=Acinetobacter baumannii (strain ATCC 17978 / CIP 53.77 / LMG 1025 / NCDC KC755 / 5377) GN=A1S_0836 PE=4 SV=1 - [A3M2X6_ACIBT]	0,51	Cytoplasm
A3M268	Putative NAD(P)-binding enzyme OS=Acinetobacter baumannii (strain ATCC 17978 / CIP 53.77 / LMG 1025 / NCDC KC755 / 5377) GN=A1S_0559 PE=4 SV=2 - [A3M268_ACIBT]	0,52	Inner membrane
A3M1Y5	Putative ferric siderophore receptor protein OS=Acinetobacter baumannii (strain ATCC 17978 / CIP 53.77 / LMG 1025 / NCDC KC755 / 5377) GN=A1S_0474 PE=3 SV=1 - [A3M1Y5_ACIBT]	0,52	Outer membrane
A3M2F0	Uncharacterized protein OS=Acinetobacter baumannii (strain ATCC 17978 / CIP 53.77 / LMG 1025 / NCDC KC755 / 5377) GN=A1S_0645 PE=4 SV=2 - [A3M2F0_ACIBT]	0,52	Secreted
A3M140	ATP synthase subunit b OS=Acinetobacter baumannii (strain ATCC 17978 / CIP 53.77 / LMG 1025 / NCDC KC755 / 5377) GN=atpF PE=3 SV=2 - [ATPF_ACIBT]	0,52	Inner membrane
A3M3P2	Uncharacterized protein OS=Acinetobacter baumannii (strain ATCC 17978 / CIP 53.77 / LMG 1025 / NCDC KC755 / 5377) GN=A1S_1106 PE=4 SV=1 - [A3M3P2_ACIBT]	0,52	Secreted
A3M3U3	Uncharacterized protein OS=Acinetobacter baumannii (strain ATCC 17978 / CIP 53.77 / LMG 1025 / NCDC KC755 / 5377) GN=A1S_1157 PE=4 SV=2 - [A3M3U3_ACIBT]	0,52	Cytoplasm
A3M3E9	Urease accessory protein UreD OS=Acinetobacter baumannii (strain ATCC 17978 / CIP 53.77 / LMG 1025 / NCDC KC755 / 5377) GN=ureD PE=3 SV=2 - [URED_ACIBT]	0,52	Cytoplasm

A3M146	Uncharacterized protein OS=Acinetobacter baumannii (strain ATCC 17978 / CIP 53.77 / LMG 1025 / NCDC KC755 / 5377) GN=A1S_0157 PE=4 SV=2 - [A3M146_ACIBT]	0,52	Periplasm
A3M5D5	Dihydrolipoamide dehydrogenase OS=Acinetobacter baumannii (strain ATCC 17978 / CIP 53.77 / LMG 1025 / NCDC KC755 / 5377) GN=A1S_1702 PE=4 SV=1 - [A3M5D5_ACIBT]	0,54	Cytoplasm
A3M976	50S ribosomal protein L29 OS=Acinetobacter baumannii (strain ATCC 17978 / CIP 53.77 / LMG 1025 / NCDC KC755 / 5377) GN=rpmC PE=3 SV=1 - [RL29_ACIBT]	0,54	Cytoplasm
A3M615	Uncharacterized protein OS=Acinetobacter baumannii (strain ATCC 17978 / CIP 53.77 / LMG 1025 / NCDC KC755 / 5377) GN=A1S_1932 PE=4 SV=1 - [A3M615_ACIBT]	0,54	Inner membrane
A3M4U6	Putative membrane protein OS=Acinetobacter baumannii (strain ATCC 17978 / CIP 53.77 / LMG 1025 / NCDC KC755 / 5377) GN=A1S_1513 PE=3 SV=2 - [A3M4U6_ACIBT]	0,55	Cytoplasm
A3M1W4	Putative biopolymer transport protein (ExbB) OS=Acinetobacter baumannii (strain ATCC 17978 / CIP 53.77 / LMG 1025 / NCDC KC755 / 5377) GN=A1S_0453 PE=3 SV=2 - [A3M1W4_ACIBT]	0,56	Inner membrane
A3M2C9	Putative lipoprotein OS=Acinetobacter baumannii (strain ATCC 17978 / CIP 53.77 / LMG 1025 / NCDC KC755 / 5377) GN=A1S_0624 PE=3 SV=2 - [A3M2C9_ACIBT]	0,56	Periplasm
A3M3B9	Ferric enterobactin receptor OS=Acinetobacter baumannii (strain ATCC 17978 / CIP 53.77 / LMG 1025 / NCDC KC755 / 5377) GN=A1S_0981 PE=3 SV=1 - [A3M3B9_ACIBT]	0,57	Outer membrane
A3M7D6	Oxidoreductase short-chain dehydrogenase/reductase family OS=Acinetobacter baumannii (strain ATCC 17978 / CIP 53.77 / LMG 1025 / NCDC KC755 / 5377) GN=A1S_2411 PE=4 SV=2 - [A3M7D6_ACIBT]	0,57	Cytoplasm
A3M472	Putative VGR-related protein OS=Acinetobacter baumannii (strain ATCC 17978 / CIP 53.77 / LMG 1025 / NCDC KC755 / 5377) GN=A1S_1288 PE=4 SV=1 - [A3M472_ACIBT]	0,58	Cytoplasm
A3M604	Ferrichrome-iron receptor OS=Acinetobacter baumannii (strain ATCC 17978 / CIP 53.77 / LMG 1025 / NCDC KC755 / 5377) GN=A1S_1921 PE=3 SV=2 - [A3M604_ACIBT]	0,58	Outer membrane
A3M3V8	Putative transposase OS=Acinetobacter baumannii (strain ATCC 17978 / CIP 53.77 / LMG 1025 / NCDC KC755 / 5377) GN=A1S_1172 PE=4 SV=2 - [A3M3V8_ACIBT]	0,58	Cytoplasm
A3M9R8	Putative peptide signal OS=Acinetobacter baumannii (strain ATCC 17978 / CIP 53.77 / LMG 1025 / NCDC KC755 / 5377) GN=A1S_3273 PE=4 SV=1 - [A3M9R8_ACIBT]	0,58	Secreted
A3M3R8	Oxidoreductase short chain dehydrogenase/reductase family OS=Acinetobacter baumannii (strain ATCC 17978 / CIP 53.77 / LMG 1025 / NCDC KC755 / 5377) GN=A1S_1132 PE=3 SV=2 - [A3M3R8_ACIBT]	0,59	Cytoplasm
A3M2V3	ATP-dependent dethiobiotin synthetase BioD OS=Acinetobacter baumannii (strain ATCC 17978 / CIP 53.77 / LMG 1025 / NCDC KC755 / 5377) GN=bioD PE=3 SV=2 - [A3M2V3_ACIBT]	0,59	Cytoplasm
A7FAX8	Uncharacterized protein OS=Acinetobacter baumannii (strain ATCC 17978 / CIP 53.77 / LMG 1025 / NCDC KC755 / 5377) GN=A1S_3541 PE=4 SV=2 - [A7FAX8_ACIBT]	0,59	Cytoplasm

A3M5R8	Aldehyde dehydrogenase OS=Acinetobacter baumannii (strain ATCC 17978 / CIP 53.77 / LMG 1025 / NCDC KC755 / 5377) GN=A1S_1835 PE=4 SV=2 - [A3M5R8_ACIBT]	0,60	Cytoplasm
A3M2E9	Uncharacterized protein OS=Acinetobacter baumannii (strain ATCC 17978 / CIP 53.77 / LMG 1025 / NCDC KC755 / 5377) GN=A1S_0644 PE=4 SV=2 - [A3M2E9_ACIBT]	0,60	Mitochondrion
A3M1E1	Alginate biosynthesis protein OS=Acinetobacter baumannii (strain ATCC 17978 / CIP 53.77 / LMG 1025 / NCDC KC755 / 5377) GN=A1S_0260 PE=4 SV=2 - [A3M1E1_ACIBT]	0,60	Inner membrane
A7FAX2	Uncharacterized protein OS=Acinetobacter baumannii (strain ATCC 17978 / CIP 53.77 / LMG 1025 / NCDC KC755 / 5377) GN=A1S_3535 PE=4 SV=2 - [A7FAX2_ACIBT]	0,60	Periplasm
A3M6C3	Putative phage integrase OS=Acinetobacter baumannii (strain ATCC 17978 / CIP 53.77 / LMG 1025 / NCDC KC755 / 5377) GN=A1S_2040 PE=4 SV=2 - [A3M6C3_ACIBT]	0,60	Mitochondrion
A7FB06	Uncharacterized protein OS=Acinetobacter baumannii (strain ATCC 17978 / CIP 53.77 / LMG 1025 / NCDC KC755 / 5377) GN=A1S_3569 PE=4 SV=2 - [A7FB06_ACIBT]	0,60	Cytoplasm

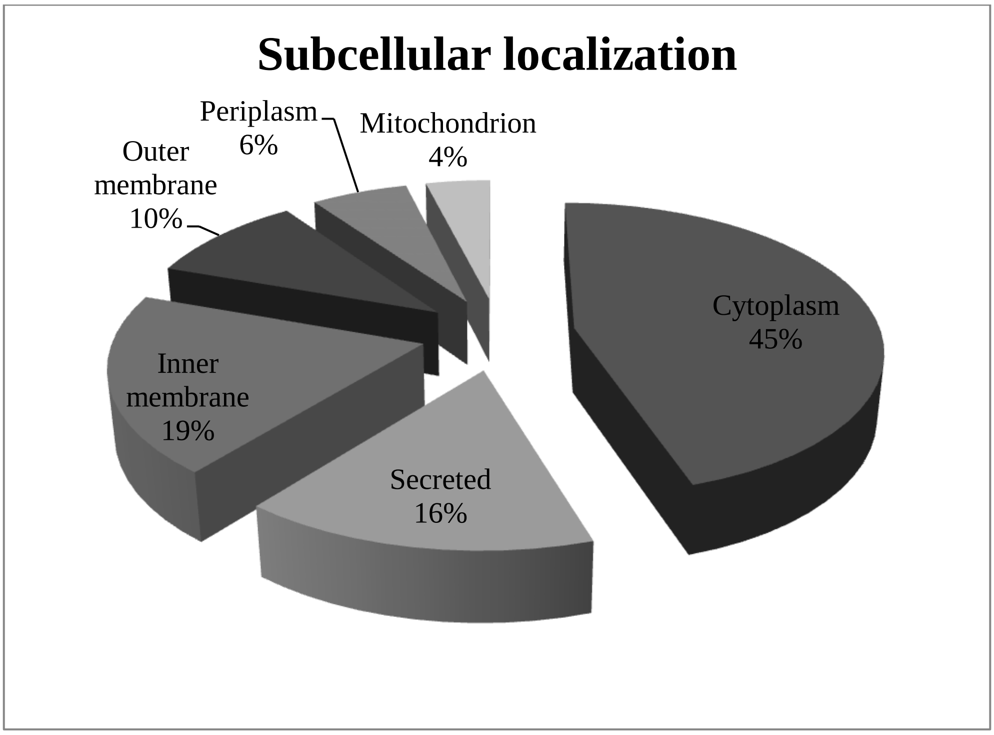

Figure S1. Subcellular localization of subexpressed proteins under hypoxia (1%O_2).
